# Plasma preparation to measure FDA-approved protein markers by selected reaction monitoring

**DOI:** 10.1186/s40169-015-0071-4

**Published:** 2015-10-15

**Authors:** Olga I. Kiseleva, Yulia A. Romashova, Natalia E. Moskaleva, Natalia A. Petushkova, Nadezhda B. Teryaeva, Artem Yu. Belyaev, Andrey V. Lisitsa

**Affiliations:** Orekhovich Institute of Biomedical Chemistry, Pogodinskaya str. 10/8, 119121 Moscow, Russia; Burdenko Neurosurgery Institute, 4th Tverskaya-Yamskaya str. 16, 125047 Moscow, Russia

**Keywords:** Proteomics, Biomarkers, Mass spectrometry, Selected reaction monitoring

## Abstract

**Background:**

The development of commercially available panels for human blood plasma screening via selected reaction monitoring (SRM) offers reliable, cost-efficient and highly-standardized discovery and validation of protein biomarkers. However, protein detection by SRM can be hampered by interfering peptide fragment ions. To estimate the influence of interference on protein detection, we performed different types of sample preparation and implemented SRM measurements for well-characterized protein targets approved by the US Food and Drug Administration.

**Methods:**

We used the PlasmaDeepDive™ SRM assay from BiognoSYS AG for absolute quantification of 18 proteins in 19 samples of human plasma using three different protocols for sample preparation. SRM measurements were performed using iRT standards for retention time normalization and isotopically-labeled reference peptides for absolute quantification. SpectroDive™ software was used for automated detection of reliable peak groups.

**Results:**

Fourteen targeted proteins were quantitatively measured in more than half of the samples. Depletion of highly-abundant plasma proteins and peptide fraction clean-up on centrifuge plates resulted in detection of all 18 targeted proteins in femtomolar to picomolar concentrations.

**Conclusions:**

It was shown that commercially designed SRM kits are suitable for SRM detection of well-established plasma/serum biomarkers.

**Electronic supplementary material:**

The online version of this article (doi:10.1186/s40169-015-0071-4) contains supplementary material, which is available to authorized users.

## Background

Selected Reaction Monitoring (SRM) is a targeted mass spectrometry method which has emerged as a promising challenger to antibody techniques of protein analysis in biological samples [1]. SRM assay development is more cost-effective than immunoaffinity approaches. The process of assay development requires synthesizing analytical quantities of isotopically-labeled peptides and determining the unique pairs of transitions between parent and product ions of proteotypic peptides with the amino acid sequence non-recurrent in other proteins [2].

The detection method, based on triple quadrupole mass spectrometry, isolates the proteotypic peptide by its *m/z* and quantifies the content of fragments in a sample. This physical principle of signal registration of characteristic ions’ pairs ensures high selectivity and sensitivity of SRM when analyzing low-abundance proteins [3]. It also should be noted that SRM analysis requires only a few microliters of blood plasma [4].

The selection of interference-free proteotypic peptides is the main obstacle to SRM assay development. Due to its technical limitations, the quadrupole mass spectrometer cannot precisely isolate charged particles of particular *m/z*, which results in overlapping flows of charged particles originating from different analytes within the given *m/z* window that cannot be resolved because of their similar peptidic natures [5]. This interference can distort mass spectrometric signatures of peptides in the complex matrix of a biological sample. Signal distortion hampers the utility of SRM measurement for quantitative analysis. For example, because of interference when developing SRM assays for 1000 possible cancer protein targets, Huttenhain et al. could only identify a few dozen proteins with non-interfering proteotypic peptides in depleted plasma [6].

Companies specializing in the development of commercial kits of peptides for SRM analysis have emerged in the market. Peptides not interfering with components of the biological matrix as well as peptides used in retention time calibration are prerequisites for commercial kits. Commercial kits are typically delivered with specialized software automating data processing such as Skyline (MacCoss Lab of Biological Mass Spectrometry, University of Washington, Seattle, WA, USA) [7], which does not require the studying of complex software documentation and is focused on assay development and subsequent results analysis in “plug-and-play” mode.

In this study we used a commercially available SRM kit to analyze the proteotypic peptides of proteins approved by the US Food and Drug Administration (FDA) as plasma/serum biomarkers [8]. Plasma samples were prepared according to different protocols in order to analyze the effect of sample preparation on interference of proteotypic peptides.

## Methods

We studied 18 proteins in 19 blood plasma samples from patients aged 50–70 years. The samples were obtained with informed consent of the patients within the framework of clinical research approved by the Ethics Committee of N. N. Burdenko Neurosurgery Institute, Moscow, Russia.

Plasma samples were stored at −80 °C without refreezing. The PlasmaDeepDive™ (BiognoSYS AG, Zurich, Switzerland) kit containing a mixture of isotopically-labeled peptide standards was used to perform SRM measurements of 100 proteins (one proteotypic peptide for each protein), of which 18 were the subject matter of our research. The sample preparation including protein reduction, alkylation and tryptic cleavage was performed according to the PlasmaDeepDive™ kit manufacturer’s directions [9] using three different protocols. In the first protocol, crude plasma samples were subjected to tryptic cleavage (Series #1). In the second protocol (Series #2), removal of buffer components and sample desalting were accomplished using centrifugal MACROSpin plates (Nest Group, Southborough, MA, USA). In the third protocol (Series #3), the process of depletion was carried out using a ProteoPrep^®^ Immunoaffinity Albumin and IgG Depletion Kit (Sigma-Aldrich, St. Louis, MO, USA), followed by trypsinolysis and peptide extraction on MACROSpin centrifuge plates.

Protein digestion was performed using trypsin (0.4 µg/µl, Promega, Madison, WI, USA) in darkness at a temperature of 37 °C for 3 h with stirring. The ratio of trypsin:protein was 1:100. The reaction was stopped by adding 20 % trifluoroacetic acid solution (Acros Organics, Morris Plains, NJ, USA), adjusting the pH of the peptide solution to 2.0. The trypsinolyzed plasma was dried, redissolved in liquid chromatography solution containing 0.1 % formic acid in water, and spiked with isotopically-labeled peptide standards from the PlasmaDeepDive™ kit. The UltiMate^®^ 3000 RSLCnano Standard LC system (Dionex, Sunnyvale, CA, USA) in tandem with the mass spectrometry detector TSQ Vantage (Thermo Scientific, Waltham, MA, USA) equipped with an ionization source Easy-Spray was used for chromatographic mass spectrometry analysis. Mobile phase A was 1 % acetonitrile (AcN)/0.1 % formic acid (FA)/H_2_O, phase B was H_2_O/0.1 % FA/AcN, and the flow rate was 0.3 μl/min. The percentage of mobile phase B was increased in a gradient from 5 to 35 % over 35 min, then increased to 99 % in the next 5 min and maintained there for 10 min.

The volume of the peptide compound injection was 3 μl, using a flow rate of 0.3 ml/min. The voltage on the electrode was 1900 V with the capillary temperature of 200 °C. The impact energy values were set in accordance with the SRM kit preferences [9].

The raw files obtained were processed by SpectroDive™ and Skyline programs. Transition lists were imported from SpectroDive™ into Skyline to generate.sky and .skyd files to place into PASSEL (ID PASS00633) [10] and provide access to SRM spectra in a publicly-available format. Retention time recalibration using iRT-peptide [11] characteristic peaks was done automatically when loading spectra in SpectroDive™. The quality of peptide detection was monitored by the q value calculated by mQuest/mProphet software [12]. A peptide was considered to be reliably detected at a q value ≤0.01.

## Results and discussion

**Fig. 1:**
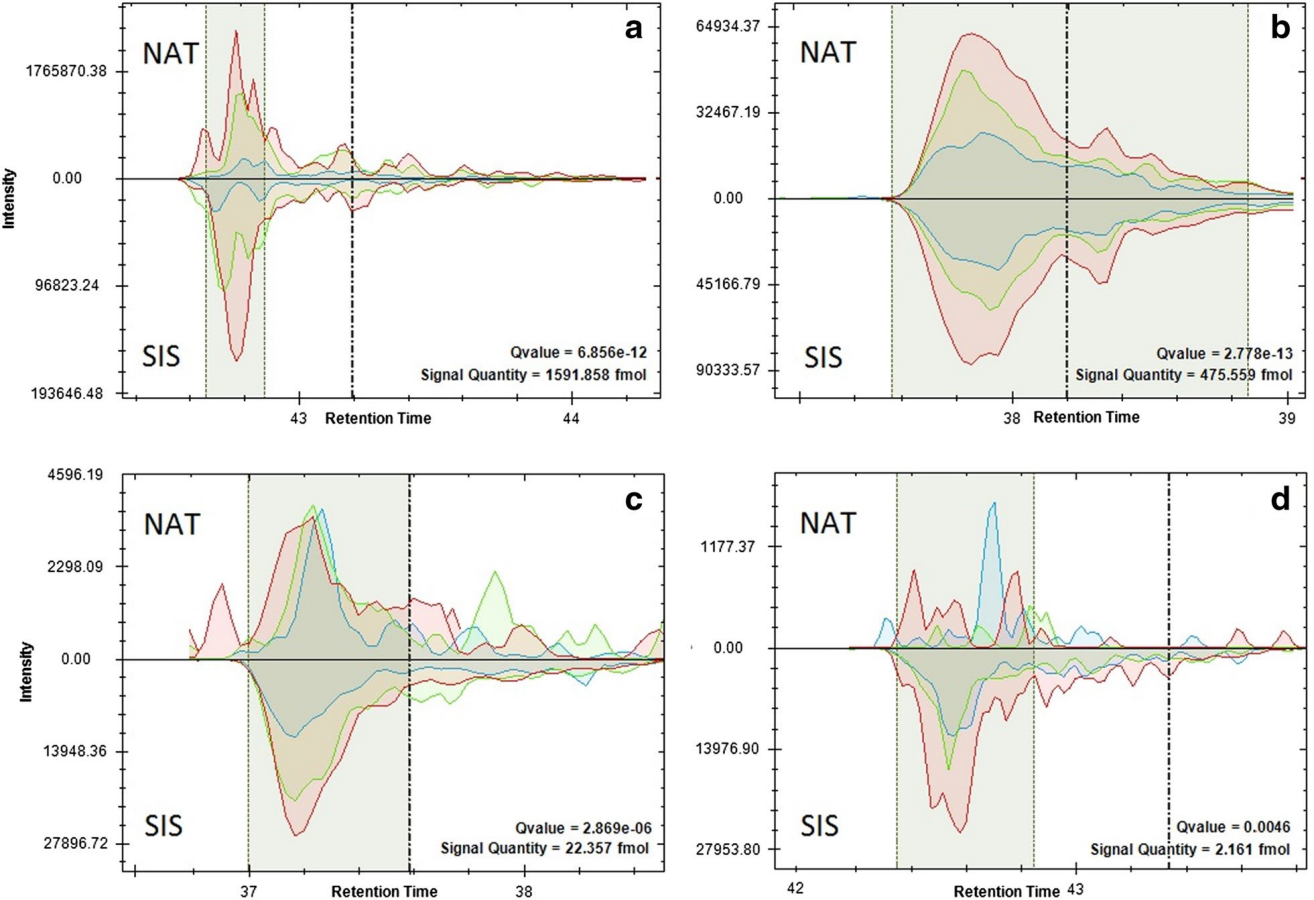
Extracted ion chromatograms of native and isotopically-labeled proteotypic peptides (marked as “NAT” and “SIS”, respectively) visualized with SpectroDive™ software for qualitative and quantitative analysis using three transitions per peptide: **a** SVLGQLGITK (Alpha 1-antitrypsin, P01009), high concentration; **b** GGYTLVSGYPK (Hemopexin, P02790), moderate concentration; **c** GYTQQLAFR (Complement C3, P01024), low concentration; **d** AVSPLPYLR (von Willebrand factor, P04275), in traces. *Shaded zones* indicate peak integration boundaries. *Dashed lines* mark peptide elution time predicted by iRT-calibration

A total of 190 SRM spectra were acquired; original raw files and respective transitions data in the.sky and .skyd format were placed in PASSEL. The spectra were allocated into the three protocols of sample preparation. Figure 1 shows examples of SRM spectra in the SpectroDive™ program; Additional file 1: Figure S1 shows the same results in Skyline. Following the PlasmaDeepDive™ protocol, three transitions per peptide were used for detection. Examples of peptides at different concentrations ranging from 2 μM (Fig. 1a) to 1 fM (Fig. 1d) are given for illustration.

High intensity (1.6 × 10^6^, Fig. 1a) transitions of the target peptide registered in a biological sample showed some distortions where transition peaks were split. Peak distortion was observed for the isotopically-labeled standard as well. Peak distortion for an endogenous peptide and spike-in peptide standard was presumably caused by specific features of the chromatographic elution, since approximately half of the cases measuring the corresponding transition peaks had similar aberrations both for native and isotopically-labeled peptides. Despite imperfect chromatographic conditions, error assessment for this group of peaks was low (q value <10^−11^); therefore, the peptide was considered to be detected, and its content evaluation verifiable.

Figure 1b shows an example of unambiguous peptide detection. The shapes of fragment ion peaks for targeted and reference peptides coincide almost perfectly. In this case, the ratio of the transitions intensity for both the heavy and light peptides was maintained throughout the elution interval. It should be noted that in Fig. 1b, the ratio of the content of endogenous peptide to the reference peptide was 1:1.5, in contrast to Fig. 1a, where the proportion was 10:1. Another situation is presented by the chromatograms of peptides in low concentration (Fig. 1c), where the peak group for endogenous peptide was corrupted due to interference. The most questionable case of peptide detection is shown in Fig. 1d. The intensity of targeted peptide did not exceed 1500. The shaded area indicates the interval in which several peaks of the two most intensive transitions were observed. However, the q value assessment for the peaks group shown in Fig. 1d still fell below the accepted threshold of q ≤ 0.01, which allowed us to consider this and a few other similar situations as borderline but acceptable for quantitative evaluation.

The summary of all acquired SRM spectra with q values ≤0.01 is given in Additional file 2: Table S1. The number of samples in which targeted proteins (peptides) were detected in one, two or three technical runs was counted; some examples are shown in Table 1. For example, apolipoprotein A1 peptide was detected in each of the 19 samples in all technical runs of each sample preparation; i.e., detection was observed in a total of 19 × 3 = 57 chromatograms. Serum albumin was detected in 100 % of the cases in the first two series (i.e., in all samples in every technical run), while in the third series protein detection was achieved only in two samples and in two technical runs. In that same third series, von Willebrand factor was detected in only four samples of one technical run, and in two of those samples detection was reliable in two runs only.

**Table 1:** Examples of protein detection in 19 plasma samples using the PlasmaDeepDive™ kit and SpectroDive™ software with a q value <0.01

Protein	Reproducibility of detection in all series, %	Series #1				Series #2				Series #3			
		Total^a^	Number of tech. runs			Total^a^	Number of tech. runs			Total^a^	Number of tech. runs		
			1	2	3		1	2	3		1	2	3
Apolipoprotein A1	100	57			19	57			19	57			19
Serum albumin	99	57			19	57			19	55		2	17
Insulin-like growth factor-binding protein 3	70	10	2	1	2	56		1	18	53		4	15
Cystatin-C	33	7	2	1	1	23	5	3	4	27	5	5	4
von Willebrand factor	5	0	0			1	1			8	4	2	

^a^Total: number of technical runs in which the peptide was detected, multiplied by the number of samples

Peptides of 11 FDA-approved proteins were detected in each sample from any of the sample preparation protocols in at least two technical runs. Highly abundant proteins, including the aforementioned serum albumin and apolipoprotein A1, but also fibrinogen α-chain, haptoglobin and others (Additional file 2: Table S1) belonged to this group.

Five proteins, including insulin-like growth factor II and ceruloplasmin, were not detected in any of the samples in the series of experiments performed with whole plasma. Signatures of two other proteins, cystatin C and insulin-like growth factor-binding protein III, were detected in single samples, and in only a few cases did detection occur in all three technical runs. However, two-thirds of the targeted proteins were successfully detected in almost all plasma samples in three technical runs.

Peptide extractions done in the second series of experiments allowed for detection of peptides from 17 proteins (12 of them in all samples, and 11 in all technical runs). Compared with the first series of experiments, we were able to reliably detect four proteins not previously detected in crude plasma, including von Willebrand factor, which was detected in one of the samples in a single technical run.

In the third series of experiments using immunoaffinity depletion of highly abundant plasma proteins, we were able to detect all 18 protein targets. Nine proteins were present in all samples in every technical run, while the other three proteins were present in at least two technical runs in all samples. In this series, it was possible to detect insulin-like growth factor II not identified in the first two series. This protein was quantified over 16 samples; the average concentration was 6.1 ± 0.7 fM. The depletion procedure used in the third protocol of sample preparation allowed us to estimate inter-individual variation of von Willebrand factor concentration (1.15 fM, CV = 59 %) in six samples. The rare detection of this protein is surprising given its low content in plasma, estimated as 0.1 µg/ml according to published data [13].

Peptides detected in three technical runs were selected to assess the reproducibility of quantitative measurements. The concentrations of nine out of 18 proteins were measured with CV <30 % between technical runs (Additional file 3: Figure S2). Series #1 was characterized by the highest reproducibility of quantification. In the first series, median CV was 28 %, followed by 53 and 52 % for Series #2 and Series #3, respectively. However, the differences observed were not statistically significant.

**Fig. 2:**
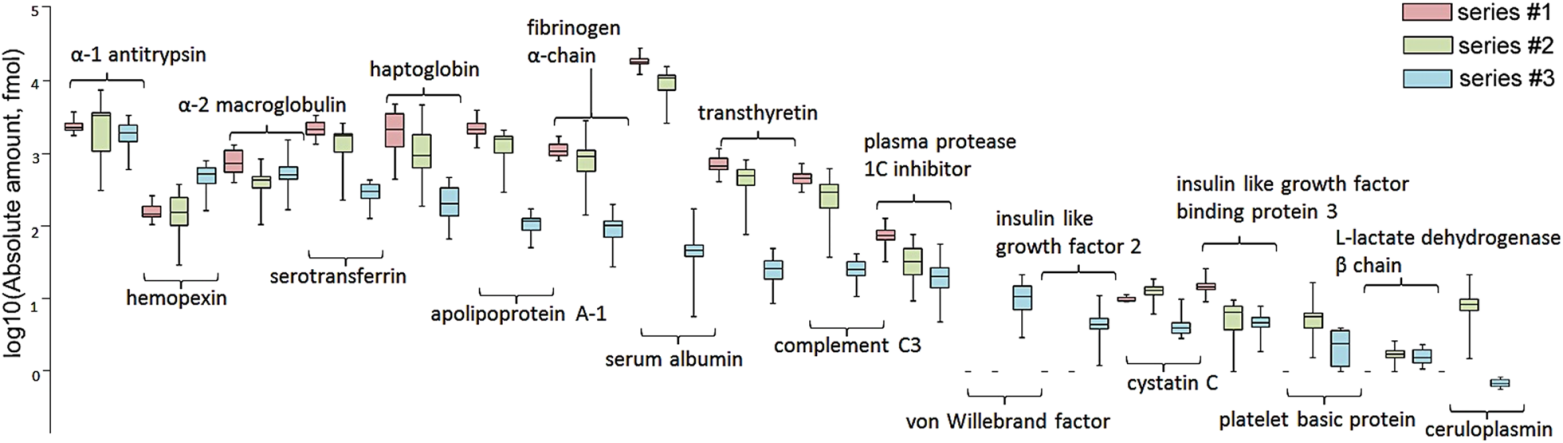
*Box plot* for concentrations of FDA-approved proteins averaged across three technical runs and 19 plasma samples using three protocols of sample preparation (Series #1–3)

Figure 2 shows target protein concentrations measured using the PlasmaDeepDive™ kit. The data were in the range of 31 pM (serum albumin, Series #1) to 0.1 fM (ceruloplasmin, Series #3). As can be seen from the box plot, the protein content was dependent on the sample preparation protocol. The difference in protein quantitative assessment may be considerable; for example, for fibrinogen α-chain in Series #1 and Series #2, a value of about 1 pmol was obtained, while in Series #3, the absolute concentration was ten times lower, evidently due to depletion.

A metaanalysis by Polanski and Anderson [14] provides data on plasma protein content, where different immunoaffinity approaches were used to determine protein concentrations. We compared the results of our Series #3 experiment with the data from this study [14]. The coefficient of determination in double logarithmic coordinates was 0.80. This high value is due to the presence of two groups of high- and average-abundance proteins. Significant correlations were not observed in the group of high-abundance proteins (Additional file 4: Figure S3).

## Conclusions

FDA-approved plasma biomarkers are feasible for SRM detection using commercially available SRM assay kits. Detection of 18 targets required analysis of 19 samples to acquire reproducible SRM spectra. It was observed that even a rigorously optimized kit suffers from interference in some cases. In this regard, application of isotopically-labeled peptides and retention time standards is essential to assure that SRM measurements are resistant to chromatographic distortions.

Extensive sample preparation enabled detection of peptide concentrations in a range covering five orders of magnitude. In a series using crude plasma five proteins remained undetected, while application of clean-up and depletion strategies reduced the number of undetected proteins to one and zero, respectively.

Despite the fact that the overall correlation of our results with published plasma protein data was high, the results of SRM measurements for particular proteins differed by the orders of magnitude. Therefore, it is necessary to validate the SRM results by selecting reference points across the concentration range [15].

Finally, integrated SpectroDive™ software, optimized for a particular assay, provides user serviceability in mass spectra signal processing and data analysis and allows permanent inner control of retention time for peptide fragments under test.

## Additional files

10.1186/s40169-015-0071-4 Extracted ion chromatograms of native and isotopically labelled proteotypic peptides (marked as “NAT” and “SIS”, respectively) obtained with Skyline software for the peptides of PlasmaDeepDive™ kit in Series #3: а SVLGQLGITK (Alpha 1-antitrypsin, P01009), 1592 fM; b GGYTLVSGYPK (Hemopexin, P02790), 475 fM; c GYTQQLAFR (Complement C3, P01024), 22 fM; d AVSPLPYLR (von Willebrand factor, P04275), 2 fM. Dashed lines indicate peak integration boundaries.
10.1186/s40169-015-0071-4 The summary of all obtained SRM spectra with q-values ≤ 0.01.
10.1186/s40169-015-0071-4 Mean values of the coefficient of variation among technical runs in different series of measurement. Each point corresponds to the proteins detected in three technical runs (nine proteins in Series #1 and Series #3, and 11 proteins in Series #2).
10.1186/s40169-015-0071-4 Comparison between protein concentration measured by SRM (Series #3) and by other approaches [14].
