# Supplementary figures and images for: Plasma preparation to measure FDA-approved protein markers by selected reaction monitoring
Source: Clin Transl Med. 2015 Oct 15;4:32. doi: 10.1186/s40169-015-0071-4 (PMC4607682; doi:10.1186/s40169-015-0071-4)

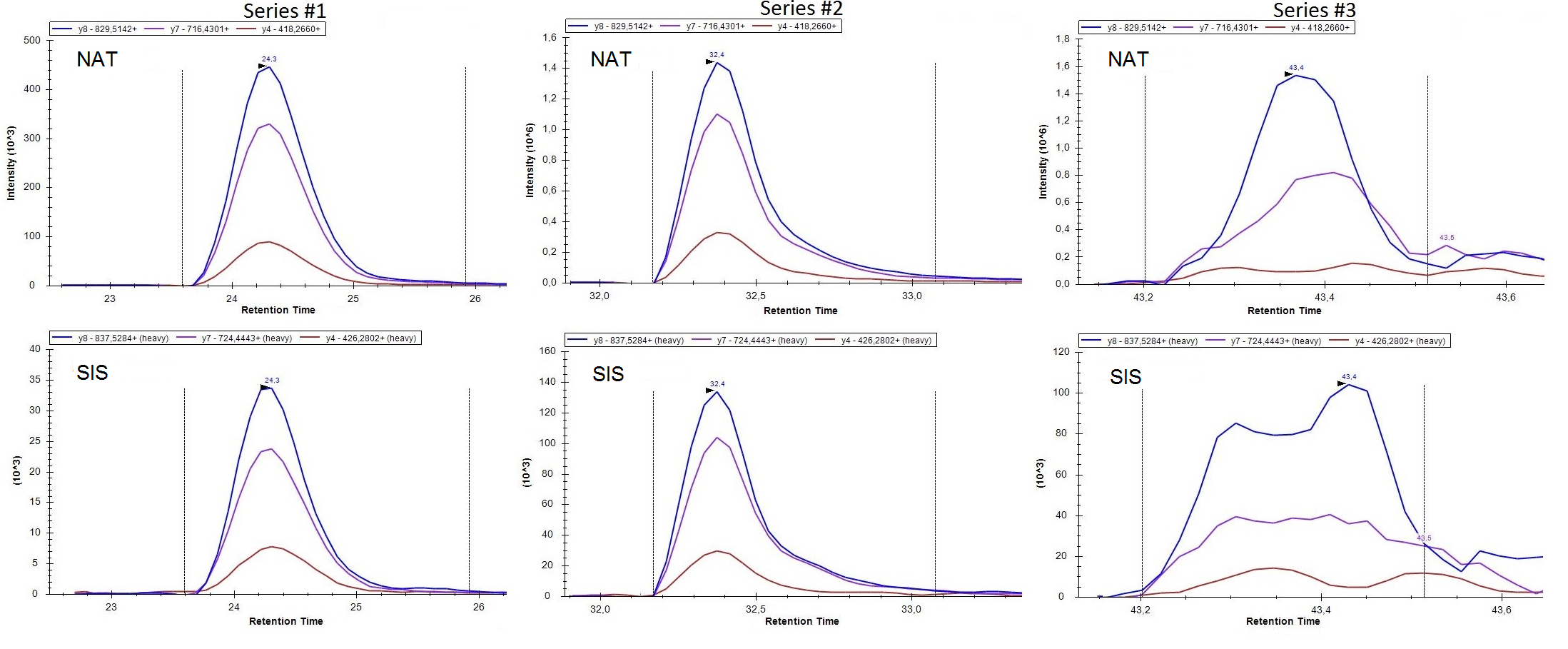

Supplement: Supplementary file 1 — 10.1186/s40169-015-0071-4 Extracted ion chromatograms of native and isotopically labelled proteotypic peptides (marked as “NAT” and “SIS”, respectively) obtained with Skyline software for the peptides of PlasmaDeepDive™ kit in Series #3: а SVLGQLGITK (Alpha 1-antitrypsin, P01009), 1592 fM; b GGYTLVSGYPK (Hemopexin, P02790), 475 fM; c GYTQQLAFR (Complement C3, P01024), 22 fM; d AVSPLPYLR (von Willebrand factor, P04275), 2 fM. Dashed lines indicate peak integration boundaries. [file 40169_2015_71_MOESM1_ESM.png]

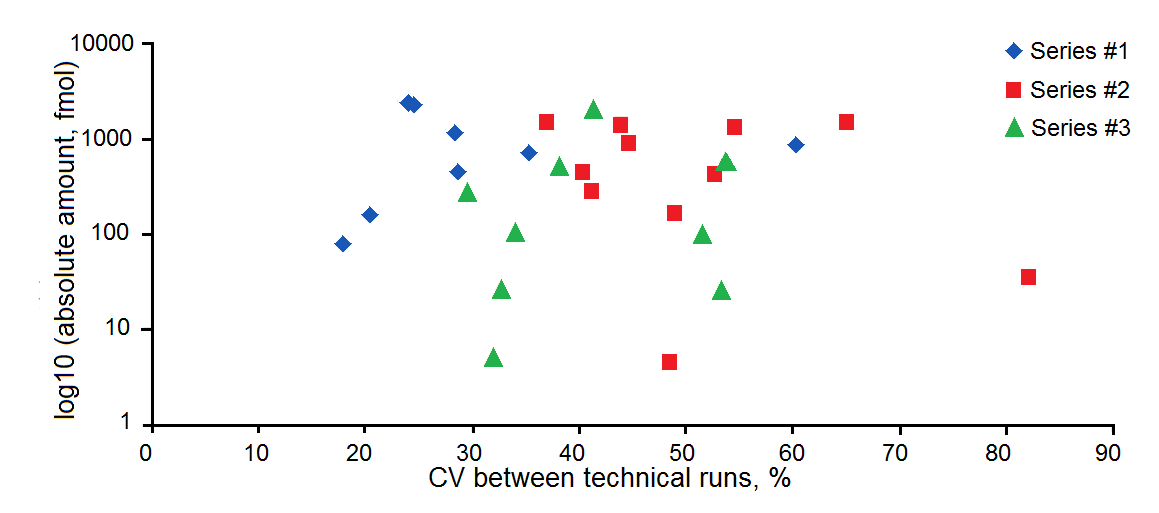

Supplement: Supplementary file 3 — 10.1186/s40169-015-0071-4 Mean values of the coefficient of variation among technical runs in different series of measurement. Each point corresponds to the proteins detected in three technical runs (nine proteins in Series #1 and Series #3, and 11 proteins in Series #2). [file 40169_2015_71_MOESM3_ESM.png]

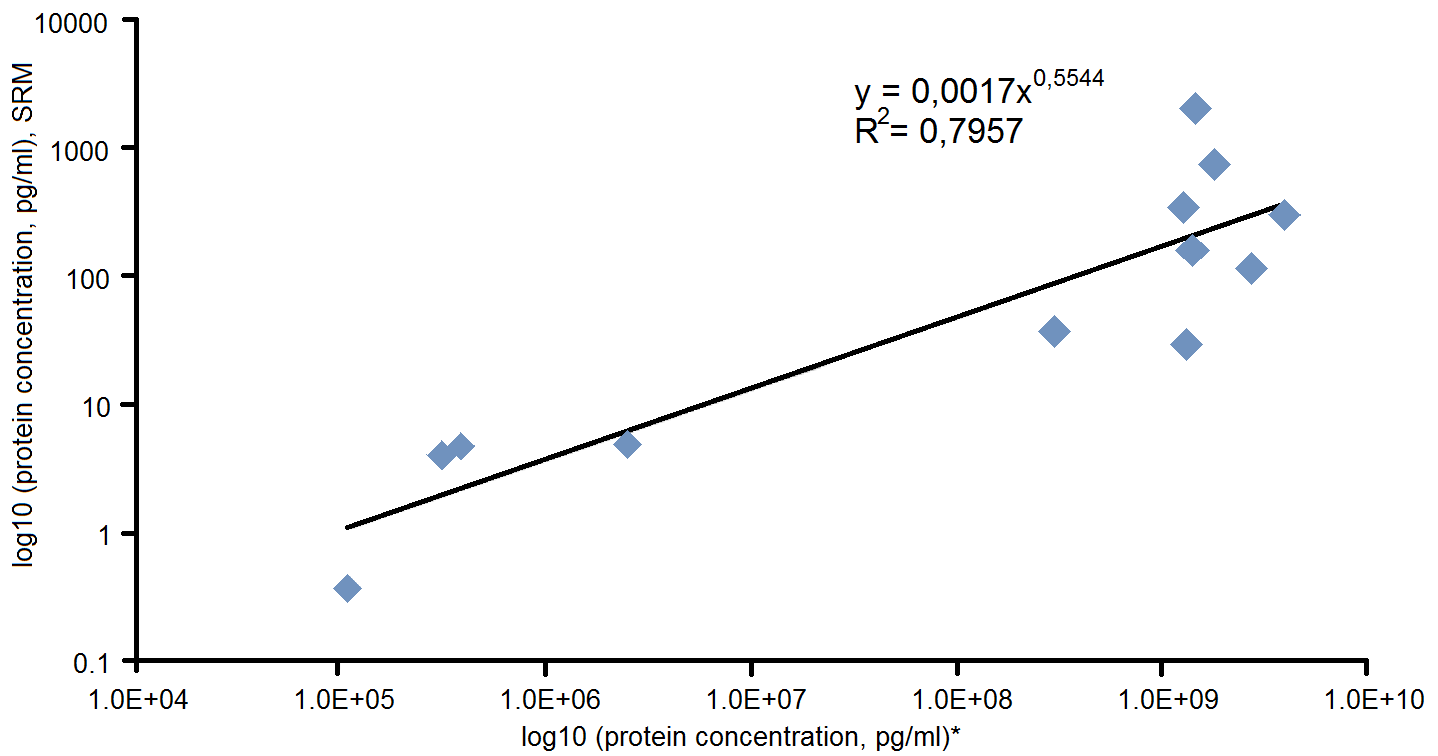

Supplement: Supplementary file 4 — 10.1186/s40169-015-0071-4 Comparison between protein concentration measured by SRM (Series #3) and by other approaches [14]. [file 40169_2015_71_MOESM4_ESM.png]
